# Supplementary material for: DMRforPairs: identifying Differentially Methylated Regions between unique samples using array based methylation profiles
Source: BMC Bioinformatics. 2014 May 15;15:141. doi: 10.1186/1471-2105-15-141 (PMC4046028; doi:10.1186/1471-2105-15-141)
Supplement: Additional file 2 — DMRforPairs output for the comparison of A431-MCF7 and NA17018-NA17105. Please start from the HTML files in each folder. Available via the BMC Bioinformatics website. [file 1471-2105-15-141-S2.zip › 1394847754114233_MOESM2_ESM/A431_MCF7/figures/27657.pdf]

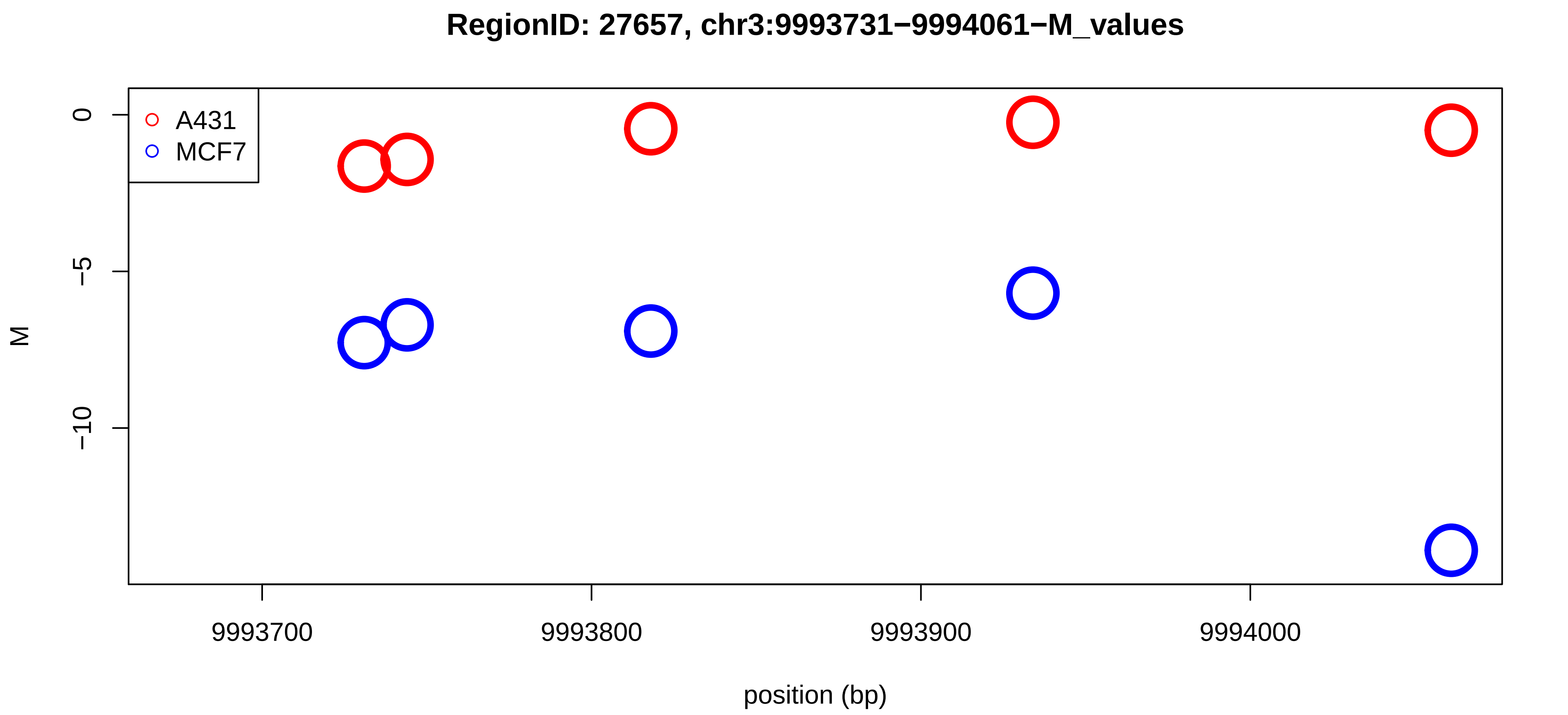

RegionID: 27657, chr3:9993731−9994061−M_values
M
0
−5
−10
A431
MCF7
9993700
9993800
9993900
9994000
position (bp)

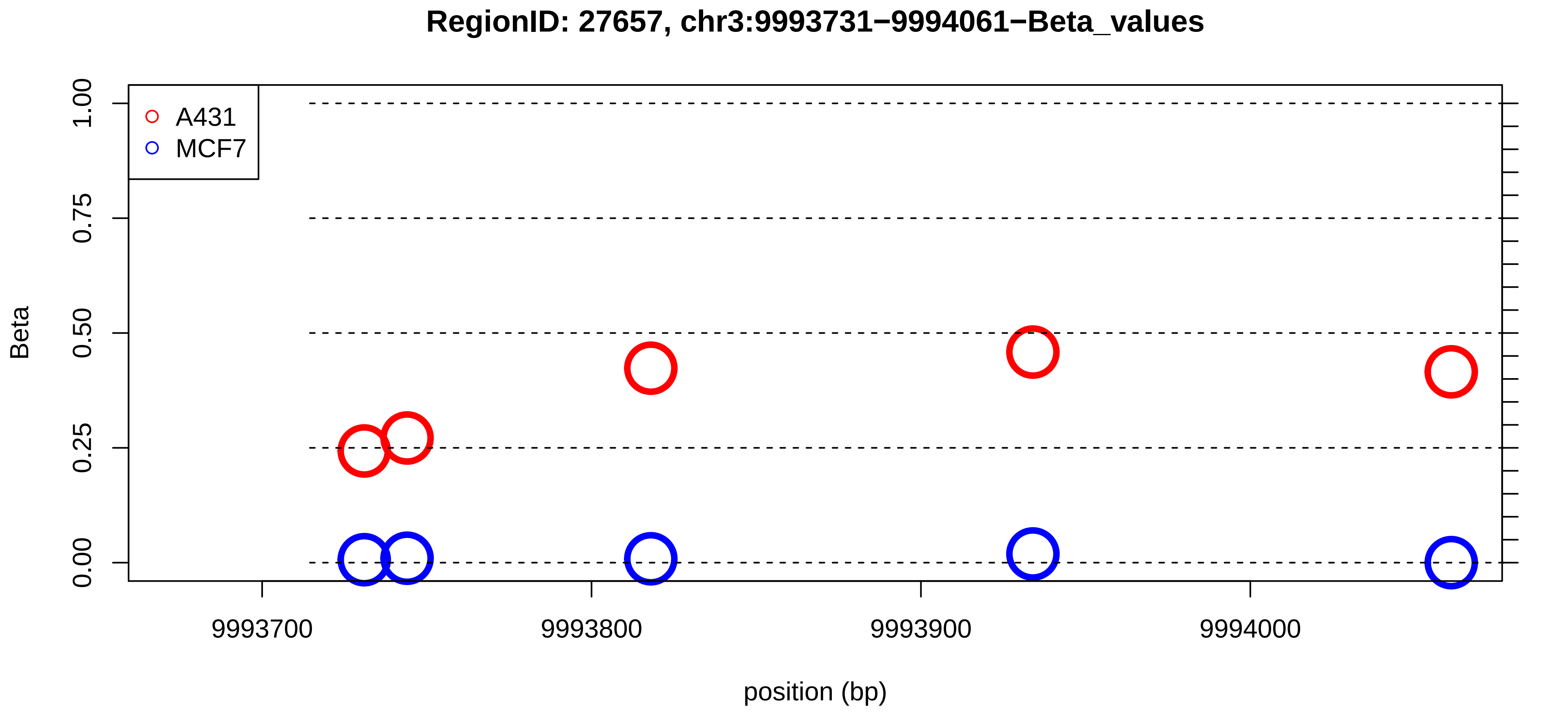

RegionID: 27657, chr3:9993731−9994061−Beta_values
Beta
1.00
0.75
0.50
0.25
0.00
A431
MCF7
9993700
9993800
9993900
9994000
position (bp)

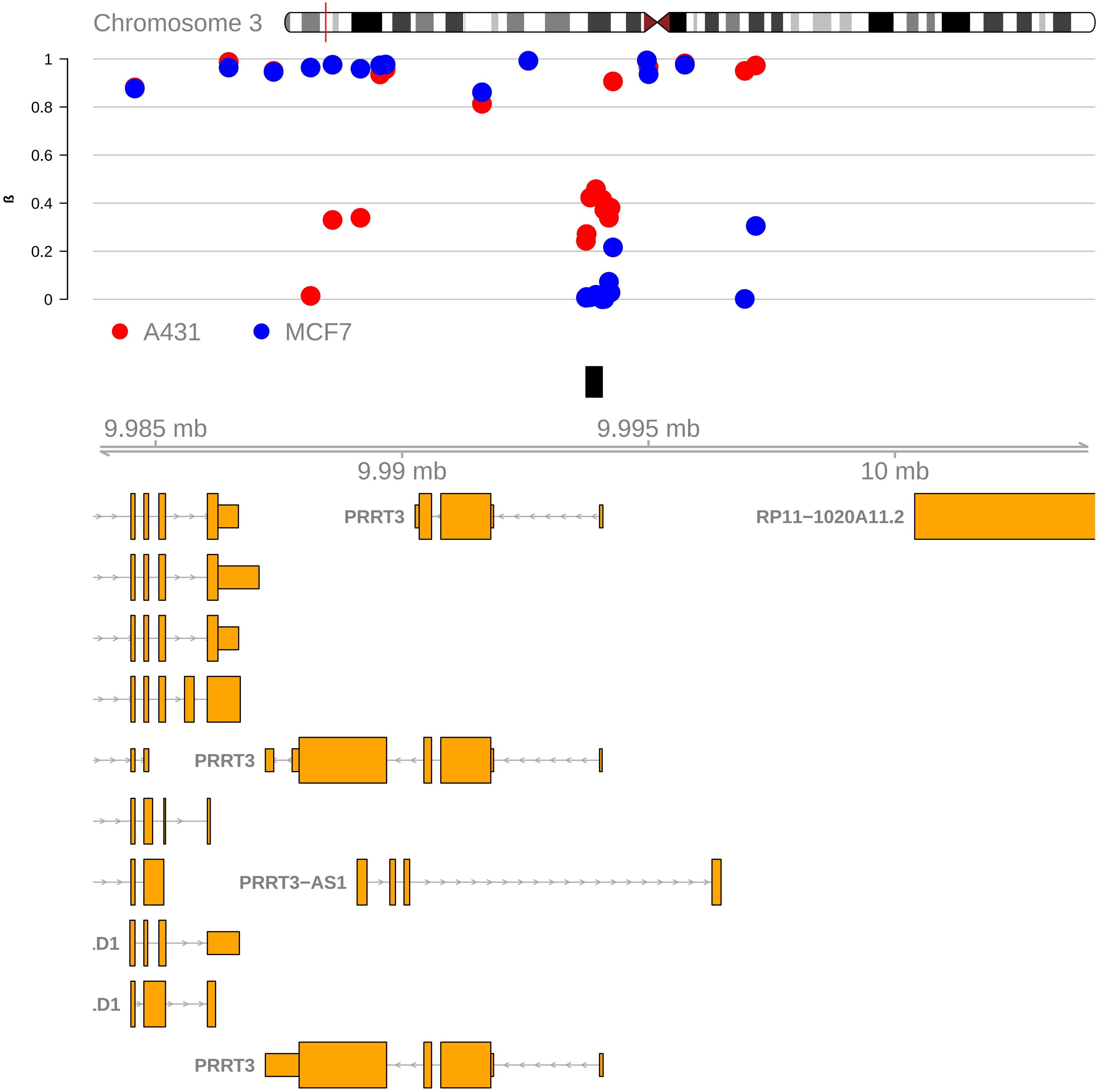

Chromosome 3
1
0.8
0.6
0.4
0.2
0
ß
A431
MCF7
9.985 mb
9.995 mb
9.99 mb
10 mb
PRRT3
RP11−1020A11.2
PRRT3
PRRT3−AS1
.D1
.D1
PRRT3
